# Supplementary material for: Influence of perceived threat of Covid-19 and HEXACO personality traits on toilet paper stockpiling
Source: PLoS One. 2020 Jun 12;15(6):e0234232. doi: 10.1371/journal.pone.0234232 (PMC7292383; doi:10.1371/journal.pone.0234232)
Supplement: S1 Table — (DOCX) [file pone.0234232.s001.docx]

**Table S1**

*Sample sizes for all residences*

| Region | *Residence* | *n* |
| --- | --- | --- |
| Europe |  |  |
|  | Austria | 15 |
|  | Belgium | 6 |
|  | France | 12 |
|  | Germany | 619 |
|  | Gibraltar | 1 |
|  | Great Britain | 21 |
|  | Greece | 2 |
|  | Ireland | 4 |
|  | Italy | 4 |
|  | Netherlands | 6 |
|  | Portugal | 1 |
|  | Romania | 1 |
|  | Russia | 1 |
|  | Serbia | 1 |
|  | Slovakia | 1 |
|  | Slovenia | 1 |
|  | Spain | 9 |
|  | Sweden | 3 |
|  | Switzerland | 19 |
|  | Ukraine | 2 |
|  |  |  |
| US/Canada |  |  |
|  | Canada | 17 |
|  | United States | 250 |
|  |  |  |
| Other |  |  |
|  | Afghanistan | 1 |
|  | Argentina | 1 |
|  | Australia | 5 |
|  | Brazil | 4 |
|  | Colombia | 2 |
|  | Egypt | 1 |
|  | Guatemala | 1 |
|  | India | 4 |
|  | Israel | 2 |
|  | Peru | 2 |
|  | Singapore | 2 |
|  | South Afrika | 1 |
|  | Turkey | 7 |
