# Supplementary material for: Influence of perceived threat of Covid-19 and HEXACO personality traits on toilet paper stockpiling
Source: PLoS One. 2020 Jun 12;15(6):e0234232. doi: 10.1371/journal.pone.0234232 (PMC7292383; doi:10.1371/journal.pone.0234232)
Supplement: S2 Table — (DOCX) [file pone.0234232.s002.docx]

**Table S2**

*English Version of the Questionnaire*

| **Item** | **Answer** |
| --- | --- |
| ###Thank you very much for your interest in this study!   In this study, we are examining the relation between different personality traits and  the consumption of toilet paper.  Completing this survey takes approximately 10 minutes.  Your data will be fully anonymized.  Thank you very much for your support!  Lisa Garbe, Richard Rau & Theo Toppe  If you have any questions, please reach out to Lisa Garbe (lisa.garbe@unisg.ch). |  |
| Next | Button |
| Voluntary agreement to participation  I hereby declare that I have understood the purpose of the survey and that I am voluntarily participating in the study.  I understand that I may choose not to participate or withdraw at any time and that withdrawal of my participation will not be penalized. |  |
| I participate in this study voluntarily. | Button |
| Please indicate to what extent the following statements apply to you. |  |
| I can look at a painting for a long time. | 5-point Likert Scale (strongly disagree to strongly agree) |
| I make sure that things are in the right spot. | 5-point Likert Scale (strongly disagree to strongly agree) |
| I remain unfriendly to someone who was mean to me. | 5-point Likert Scale (strongly disagree to strongly agree) |
| Nobody likes talking with me. | 5-point Likert Scale (strongly disagree to strongly agree) |
| I am afraid of feeling pain. | 5-point Likert Scale (strongly disagree to strongly agree) |
| I find it difficult to lie. | 5-point Likert Scale (strongly disagree to strongly agree) |
| I think science is boring. | 5-point Likert Scale (strongly disagree to strongly agree) |
| I postpone complicated tasks as long as possible. | 5-point Likert Scale (strongly disagree to strongly agree) |
| I often express criticism. | 5-point Likert Scale (strongly disagree to strongly agree) |
| I easily approach strangers. | 5-point Likert Scale (strongly disagree to strongly agree) |
| I worry less than others. | 5-point Likert Scale (strongly disagree to strongly agree) |
| I would like to know how to make lots of money in a dishonest manner. | 5-point Likert Scale (strongly disagree to strongly agree) |
| Next | Button |
| Please indicate to what extent the following statements apply to you. |  |
| I have a lot of imagination. | 5-point Likert Scale (strongly disagree to strongly agree) |
| I work very precisely. | 5-point Likert Scale (strongly disagree to strongly agree) |
| I tend to quickly agree with others. | 5-point Likert Scale (strongly disagree to strongly agree) |
| I like to talk with others. | 5-point Likert Scale (strongly disagree to strongly agree) |
| I can easily overcome difficulties on my own. | 5-point Likert Scale (strongly disagree to strongly agree) |
| I want to be famous. | 5-point Likert Scale (strongly disagree to strongly agree) |
| I like people with strange ideas. | 5-point Likert Scale (strongly disagree to strongly agree) |
| I often do things without really thinking. | 5-point Likert Scale (strongly disagree to strongly agree) |
| Even when I’m treated badly, I remain calm. | 5-point Likert Scale (strongly disagree to strongly agree) |
| I am seldom cheerful. | 5-point Likert Scale (strongly disagree to strongly agree) |
| I have to cry during sad or romantic movies. | 5-point Likert Scale (strongly disagree to strongly agree) |
| I am entitled to special treatment. | 5-point Likert Scale (strongly disagree to strongly agree) |
| Next | Button |
| How threatened do you feel by Coronavirus? | 10-point visual analogue scale (Not at all threatened to Extremely Threatened) |
| Next | Button |
| At your place of residence, are there currently any restrictions on leaving the house? | Yes/No |
| Leaving the house is only permitted in specific professions (e.g., health care) or circumstances (e.g., grocery shopping). | Yes/No |
| Leaving the house is only permitted in small groups (i.e., up to 5 persons). | Yes/No |
| At your place of residence, are there currently any restrictions on public life? | Yes/No |
| Educational facilities (Universities, Schools, Nursery schools) are closed. | Yes/No |
| Restaurants, Bars, Cafés, and similar facilities are closed. | Yes/No |
| Local public transport is restricted. | Yes/No |
| Next | Button |
| How often did you buy toilet paper in the last 14 days? | Not once  Once  Twice  Three times or more |
| How many packets of toilet paper did you buy? | None  One  Two  Three or more |
| How many rolls of toilet paper do you currently approximately have at home? | None  1 to 4  5 to 8  9 to 12  13 to 16  17 to 20  21 or more |
| The current amount of toilet paper at your house is... | Less than usual  Usual  More than usual |
| Next | Button |
| How many high-risk people are currently living in your household (e.g., due to pre-existing conditions or age)? | Indicate number |
| Is your household currently strictly self-quarantining (you are not leaving the house at all)? | Yes/No |
| For how many days have you been self-quarantining? | Indicate number |
| Next | Button |
| Gender | Female  Male  Diverse |
| Age | Indicate number |
| What is your current place of residence? | Choose from a list of all countries |
| What is your nationality? | Choose from a list of all countries |
| How many people live in your household? | Indicate number |
| In politics people sometimes talk about "left" and "right".  Where would you place yourself? | 11-point visual analog scale (Left to Right) |
| Finish survey | Button |
