# Supplementary material for: Influence of perceived threat of Covid-19 and HEXACO personality traits on toilet paper stockpiling
Source: PLoS One. 2020 Jun 12;15(6):e0234232. doi: 10.1371/journal.pone.0234232 (PMC7292383; doi:10.1371/journal.pone.0234232)
Supplement: S3 Table — (DOCX) [file pone.0234232.s003.docx]

**Table S3**

*German Version of the Questionnaire*

| **Item** | **Answer** |
| --- | --- |
| Vielen Dank für Ihr Interesse an dieser Studie!   In dieser Studie untersuchen wir den Zusammenhang zwischen bestimmten  Persönlichkeitseigenschaften und dem Konsum von Klopapier.   Das Ausfüllen dauert 5 bis 10 Minuten.  Ihre Angaben sind vollständig anonymisiert.   Vielen Dank für Ihre Unterstützung!  Lisa Garbe, Richard Rau & Theo Toppe  Bei Fragen wenden Sie sich gerne an Lisa Garbe (lisa.garbe@unisg.ch). |  |
| Weiter | Button |
| Freiwilligkeit Hiermit erkläre ich, dass ich das Vorhaben der Befragung verstanden habe und freiwillig an der Studie teilnehme.  Ich habe verstanden, dass ich jederzeit ohne Angabe von Gründen aus der Studie ausscheiden kann, ohne dass mir persönliche Nachteile entstehen. |  |
| Ich nehme freiwillig an der Befragung teil. | Button |
| Geben Sie an, wie sehr die folgenden Aussagen auf Sie zutreffen. |  |
| Ich kann mir ein Gemälde lange ansehen. | 5-point Likert Scale (Starke Ablehnung bis Starke Zustimmung) |
| Ich achte darauf, dass Dinge geordnet ablaufen. | 5-point Likert Scale (Starke Ablehnung bis Starke Zustimmung) |
| Ich bleibe abweisend, wenn jemand gemein zu mir war. | 5-point Likert Scale (Starke Ablehnung bis Starke Zustimmung) |
| Niemand redet gerne mit mir. | 5-point Likert Scale (Starke Ablehnung bis Starke Zustimmung) |
| Ich habe Angst davor Schmerzen zu empfinden. | 5-point Likert Scale (Starke Ablehnung bis Starke Zustimmung) |
| Mir fällt es schwer zu lügen. | 5-point Likert Scale (Starke Ablehnung bis Starke Zustimmung) |
| Ich finde Wissenschaft langweilig. | 5-point Likert Scale (Starke Ablehnung bis Starke Zustimmung) |
| Ich schiebe langweilige Aufgaben so lange es geht vor mir her. | 5-point Likert Scale (Starke Ablehnung bis Starke Zustimmung) |
| Ich übe häufig Kritik. | 5-point Likert Scale (Starke Ablehnung bis Starke Zustimmung) |
| Es fällt mir leicht, auf Fremde zuzugehen. | 5-point Likert Scale (Starke Ablehnung bis Starke Zustimmung) |
| Ich sorge mich weniger als andere. | 5-point Likert Scale (Starke Ablehnung bis Starke Zustimmung) |
| Ich würde gerne wissen, wie man auf unehrliche Weise viel Geld machen kann. | 5-point Likert Scale (Starke Ablehnung bis Starke Zustimmung) |
| Weiter | Button |
| Geben Sie an, wie sehr die folgenden Aussagen auf Sie zutreffen. |  |
| Ich habe viel Fantasie. | 5-point Likert Scale (Starke Ablehnung bis Starke Zustimmung) |
| Ich arbeite sehr präzise. | 5-point Likert Scale (Starke Ablehnung bis Starke Zustimmung) |
| Ich bin in der Regel schnell einer Meinung mit anderen. | 5-point Likert Scale (Starke Ablehnung bis Starke Zustimmung) |
| Ich rede gerne mit anderen. | 5-point Likert Scale (Starke Ablehnung bis Starke Zustimmung) |
| Ich kann mit Schwierigkeiten gut alleine umgehen. | 5-point Likert Scale (Starke Ablehnung bis Starke Zustimmung) |
| Ich wäre gerne berühmt. | 5-point Likert Scale (Starke Ablehnung bis Starke Zustimmung) |
| Ich mag Menschen mit sonderbaren Ideen. | 5-point Likert Scale (Starke Ablehnung bis Starke Zustimmung) |
| Ich mache oft Dinge ohne nachzudenken. | 5-point Likert Scale (Starke Ablehnung bis Starke Zustimmung) |
| Ich bleibe ruhig, auch wenn ich schlecht behandelt werde. | 5-point Likert Scale (Starke Ablehnung bis Starke Zustimmung) |
| Ich bin selten fröhlich. | 5-point Likert Scale (Starke Ablehnung bis Starke Zustimmung) |
| Ich muss bei traurigen oder romantischen Filmen weinen. | 5-point Likert Scale (Starke Ablehnung bis Starke Zustimmung) |
| Mir steht eine besondere Behandlung zu. | 5-point Likert Scale (Starke Ablehnung bis Starke Zustimmung) |
| Weiter | Button |
| Wie sehr fühlen Sie sich vom Coronavirus bedroht? | 10-point visual analogue scale (Überhaupt nicht bedroht bis Extrem bedroht) |
| Weiter | Button |
| Gibt es an Ihrem aktuellen Wohnort derzeit Ausgangsbeschränkungen? | Ja/Nein |
| Das Verlassen des Hauses ist nur für bestimmte Tätigkeiten erlaubt (z.B. bei bestimmten Berufen oder zum Einkaufen). | Ja/Nein |
| Das Verlassen des Hauses ist nur noch in kleinen Gruppen erlaubt (d.h. weniger als 5 Personen). | Ja/Nein |
| Gibt es an Ihrem Wohnort derzeit Beschränkungen des öffentlichen Lebens? | Ja/Nein |
| Öffentliche Bildungseinrichtungen sind geschlossen (Universitäten, Schulen, Kindergärten). | Ja/Nein |
| Restaurants, Bars und Cafés sind geschlossen. | Ja/Nein |
| Der öffentliche Nahverkehr ist eingeschränkt. | Ja/Nein |
| Weiter | Button |
| Wie häufig haben Sie in den letzten 14 Tagen Klopapier gekauft? | Keinmal  Einmal  Zweimal  Dreimal oder häufiger |
| Wie viele Packungen Klopapier haben Sie insgesamt gekauft? | Keine  Eine  Zwei  Drei oder mehr |
| Wie viele Rollen Klopapier haben Sie momentan schätzungsweise im Haushalt? | Keine  1 bis 4  5 bis 8  9 bis 12  13 bis 16  17 bis 20  21 oder mehr |
| Ist die aktuelle Menge an Klopapier in Ihrem Haushalt... | Weniger als gewöhnlich  Etwa gewöhnlich  Mehr als gewöhnlich |
| Weiter | Button |
| Wie viele Personen mit einem erhöhten Risiko für einen schweren Krankheitsverlauf leben in Ihrem Haushalt (z.B. aufgrund bestimmter Vorerkrankungen oder über 70 Jahre)? | Indicate number |
| Ist Ihr Haushalt momentan in einer strikten Selbst-Quarantäne (d.h. Sie verlassen das Haus gar nicht mehr)? | Ja/Nein |
| Seit wie vielen Tagen sind Sie schon in dieser strikten Selbst-Quarantäne? | Indicate number |
| Weiter | Button |
| Geschlecht | Weiblich  Männlich  Divers |
| Alter | Indicate number |
| In welchem Land leben Sie momentan? | Germany/Deutschland  Austria/Österreich  Switzerland/Schweiz |
| In welchem Bundesland leben Sie momentan? | Choose from a list of all provinces of Germany (Bundesland) |
| Welche Nationalität haben Sie? | Choose from a list of all countries |
| Wie viele Menschen leben in Ihrem Haushalt? | Indicate number |
| Man spricht in der Politik immer wieder von "links" und "rechts".  Wo würden Sie sich selbst auf dem Links-Rechts-Spektrum einordnen? | 11-point visual analog scale (Links bis Rechts) |
| Umfrage abschließen | Button |
