# Supplementary material for: Influence of perceived threat of Covid-19 and HEXACO personality traits on toilet paper stockpiling
Source: PLoS One. 2020 Jun 12;15(6):e0234232. doi: 10.1371/journal.pone.0234232 (PMC7292383; doi:10.1371/journal.pone.0234232)
Supplement: S4 Table — (DOCX) [file pone.0234232.s004.docx]

**Table S4**

*Correlations with confidence intervals*

| Variable | 1 | 2 | | 3 | | 4 | | 5 | | 6 | | 7 | | 8 | | 9 | | 10 | | 11 | | 12 | | 13 | | 14 | | 15 | 16 | | 17 | | |
| --- | --- | --- | --- | --- | --- | --- | --- | --- | --- | --- | --- | --- | --- | --- | --- | --- | --- | --- | --- | --- | --- | --- | --- | --- | --- | --- | --- | --- | --- | --- | --- | --- | --- |
|  |  |  | |  | |  | |  | |  | |  | |  | |  | |  | |  | |  | |  | |  | |  |  | |  | | |
| 1. Age |  |  | |  | |  | |  | |  | |  | |  | |  | |  | |  | |  | |  | |  | |  |  | |  | | |
|  |  |  | |  | |  | |  | |  | |  | |  | |  | |  | |  | |  | |  | |  | |  |  | |  | | |
| 2. Household size | -.04 |  | |  | |  | |  | |  | |  | |  | |  | |  | |  | |  | |  | |  | |  |  | |  | | |
|  | [-.10, .02] |  | |  | |  | |  | |  | |  | |  | |  | |  | |  | |  | |  | |  | |  |  | |  | | |
|  |  |  | |  | |  | |  | |  | |  | |  | |  | |  | |  | |  | |  | |  | |  |  | |  | | |
| 3. Quarantine duration | -.05 | -.02 | |  | |  | |  | |  | |  | |  | |  | |  | |  | |  | |  | |  | |  |  | |  | | |
|  | [-.11, .02] | [-.08, .05] | |  | |  | |  | |  | |  | |  | |  | |  | |  | |  | |  | |  | |  |  | |  | | |
|  |  |  | |  | |  | |  | |  | |  | |  | |  | |  | |  | |  | |  | |  | |  |  | |  | | |
| 4. Mobility restriction | .02 | .01 | | -.00 | |  | |  | |  | |  | |  | |  | |  | |  | |  | |  | |  | |  |  | |  | | |
|  | [-.05, .08] | [-.06, .07] | | [-.07, .06] | |  | |  | |  | |  | |  | |  | |  | |  | |  | |  | |  | |  |  | |  | | |
|  |  |  | |  | |  | |  | |  | |  | |  | |  | |  | |  | |  | |  | |  | |  |  | |  | | |
| 5. Public transport restriction | .05 | .06 | | -.02 | | .09** | |  | |  | |  | |  | |  | |  | |  | |  | |  | |  | |  |  | |  | | |
|  | [-.01, .12] | [-.00, .12] | | [-.08, .05] | | [.02, .15] | |  | |  | |  | |  | |  | |  | |  | |  | |  | |  | |  |  | |  | | |
|  |  |  | |  | |  | |  | |  | |  | |  | |  | |  | |  | |  | |  | |  | |  |  | |  | | |
| 6. Political placement | .10** | -.05 | | .09** | | -.01 | | .03 | |  | |  | |  | |  | |  | |  | |  | |  | |  | |  |  | |  | | |
|  | [.04, .17] | [-.11, .02] | | [.03, .15] | | [-.07, .05] | | [-.04, .09] | |  | |  | |  | |  | |  | |  | |  | |  | |  | |  |  | |  | | |
|  |  |  | |  | |  | |  | |  | |  | |  | |  | |  | |  | |  | |  | |  | |  |  | |  | | |
| 7. Residence | -.01 | .02 | | -.17** | | .26** | | .12** | | .09** | |  | |  | |  | |  | |  | |  | |  | |  | |  |  | |  | | |
|  | [-.08, .05] | [-.05, .08] | | [-.23, -.11] | | [.20, .31] | | [.06, .18] | | [.02, .15] | |  | |  | |  | |  | |  | |  | |  | |  | |  |  | |  | | |
|  |  |  | |  | |  | |  | |  | |  | |  | |  | |  | |  | |  | |  | |  | |  |  | |  | | |
| 8. Time size first case | -.00 | .03 | | .03 | | -.06 | | -.00 | | -.08* | | -.40** | |  | |  | |  | |  | |  | |  | |  | |  |  | |  | | |
|  | [-.07, .06] | [-.03, .09] | | [-.04, .09] | | [-.12, .00] | | [-.06, .06] | | [-.14, -.02] | | [-.45, -.35] | |  | |  | |  | |  | |  | |  | |  | |  |  | |  | | |
|  |  |  | |  | |  | |  | |  | |  | |  | |  | |  | |  | |  | |  | |  | |  |  | |  | | |
| 9. Corona | .06 | .00 | | .14** | | -.12** | | -.04 | | -.01 | | -.29** | | .06* | |  | |  | |  | |  | |  | |  | |  |  | |  | | |
|  | [-.00, .12] | [-.06, .06] | | [.08, .20] | | [-.18, -.06] | | [-.10, .02] | | [-.08, .05] | | [-.35, -.24] | | [.00, .12] | |  | |  | |  | |  | |  | |  | |  |  | |  | | |
|  |  |  | |  | |  | |  | |  | |  | |  | |  | |  | |  | |  | |  | |  | |  |  | |  | | |
| 10. ToP shopping intensity | .10** | .06 | | .05 | | .03 | | -.03 | | .02 | | -.02 | | .00 | | .08** | |  | |  | |  | |  | |  | |  |  | |  | | |
|  | [.03, .16] | [-.00, .12] | | [-.01, .11] | | [-.04, .09] | | [-.09, .03] | | [-.04, .08] | | [-.08, .04] | | [-.06, .06] | | [.02, .15] | |  | |  | |  | |  | |  | |  |  | |  | | |
|  |  |  | |  | |  | |  | |  | |  | |  | |  | |  | |  | |  | |  | |  | |  |  | |  | | |
| 11. ToP shopping frequency | .08* | .05 | | .04 | | .04 | | -.01 | | .01 | | .07* | | -.02 | | .06 | | .80** | |  | |  | |  | |  | |  |  | |  | | |
|  | [.01, .14] | [-.01, .11] | | [-.02, .11] | | [-.02, .10] | | [-.07, .05] | | [-.05, .07] | | [.00, .13] | | [-.09, .04] | | [-.00, .12] | | [.78, .82] | |  | |  | |  | |  | |  |  | |  | | |
|  |  |  | |  | |  | |  | |  | |  | |  | |  | |  | |  | |  | |  | |  | |  |  | |  | | |
| 12. ToP in household | .08* | .03 | | .09** | | -.11** | | -.06 | | .11** | | -.28** | | .06 | | .18** | | .27** | | .21** | |  | |  | |  | |  |  | |  | | |
|  | [.01, .14] | [-.04, .09] | | [.02, .15] | | [-.17, -.05] | | [-.12, .00] | | [.05, .17] | | [-.33, -.22] | | [-.01, .12] | | [.12, .24] | | [.21, .32] | | [.15, .27] | |  | |  | |  | |  |  | |  | | |
|  |  |  | |  | |  | |  | |  | |  | |  | |  | |  | |  | |  | |  | |  | |  |  | |  | | |
| 13. HH | .06 | -.01 | | .02 | | -.12** | | .04 | | .01 | | -.20** | | .03 | | .11** | | -.00 | | -.01 | | .11** | |  | |  | |  |  | |  | | |
|  | [-.00, .12] | [-.07, .05] | | [-.04, .08] | | [-.18, -.06] | | [-.03, .10] | | [-.05, .07] | | [-.26, -.14] | | [-.03, .09] | | [.05, .17] | | [-.07, .06] | | [-.08, .05] | | [.05, .17] | |  | |  | |  |  | |  | | |
|  |  |  | |  | |  | |  | |  | |  | |  | |  | |  | |  | |  | |  | |  | |  |  | |  | | |
| 14. E | -.08* | -.09** | | .02 | | .01 | | .01 | | -.06 | | -.05 | | .04 | | .20** | | .00 | | .03 | | .04 | | .04 | |  | |  |  | |  | | |
|  | [-.14, -.01] | [-.15, -.03] | | [-.04, .08] | | [-.05, .07] | | [-.05, .07] | | [-.12, .00] | | [-.11, .01] | | [-.03, .10] | | [.14, .26] | | [-.06, .06] | | [-.03, .09] | | [-.02, .10] | | [-.02, .10] | |  | |  |  | |  | | |
|  |  |  | |  | |  | |  | |  | |  | |  | |  | |  | |  | |  | |  | |  | |  |  | |  | | |
| 15. X | -.01 | .05 | | .01 | | .01 | | .01 | | -.01 | | .11** | | -.03 | | -.06 | | .02 | | .02 | | -.04 | | -.02 | | -.11** | |  |  | |  | | |
|  | [-.07, .05] | [-.01, .12] | | [-.05, .08] | | [-.05, .07] | | [-.06, .07] | | [-.07, .05] | | [.05, .17] | | [-.09, .03] | | [-.12, .00] | | [-.04, .08] | | [-.04, .09] | | [-.10, .02] | | [-.08, .04] | | [-.17, -.05] | |  |  | |  | | |
|  |  |  | |  | |  | |  | |  | |  | |  | |  | |  | |  | |  | |  | |  | |  |  | |  | | |
| 16. A | -.08* | .05 | | -.07* | | .00 | | -.02 | | .05 | | -.05 | | .02 | | -.02 | | -.01 | | -.03 | | .02 | | .14** | | -.12** | | .15** |  | |  | | |
|  | [-.14, -.01] | [-.01, .11] | | [-.13, -.00] | | [-.06, .06] | | [-.09, .04] | | [-.01, .12] | | [-.11, .01] | | [-.04, .08] | | [-.08, .04] | | [-.07, .05] | | [-.09, .03] | | [-.04, .08] | | [.08, .20] | | [-.18, -.06] | | [.09, .21] |  | |  | | |
|  |  |  | |  | |  | |  | |  | |  | |  | |  | |  | |  | |  | |  | |  | |  |  | |  | | |
| 17. C | .01 | -.02 | | .02 | | -.00 | | .04 | | .04 | | -.12** | | .02 | | -.00 | | .06* | | .05 | | .10** | | .28** | | -.05 | | .02 | .06 | |  | | |
|  | [-.06, .07] | [-.09, .04] | | [-.04, .09] | | [-.07, .06] | | [-.02, .10] | | [-.02, .10] | | [-.18, -.06] | | [-.04, .08] | | [-.06, .06] | | [.00, .13] | | [-.01, .11] | | [.04, .16] | | [.22, .33] | | [-.11, .01] | | [-.04, .08] | [-.00, .12] | |  | | |
|  |  |  | |  | |  | |  | |  | |  | |  | |  | |  | |  | |  | |  | |  | |  |  | |  | | |
| 18. O | .06* | -.00 | | .02 | | .04 | | .04 | | -.08* | | .09** | | -.02 | | -.06 | | .04 | | .01 | | -.10** | | -.06* | | -.02 | | .18** | -.04 | | -.01 | | |
|  | [.00, .13] | [-.06, .06] | | [-.04, .09] | | [-.02, .10] | | [-.02, .10] | | [-.14, -.01] | | [.03, .15] | | [-.08, .04] | | [-.12, .01] | | [-.02, .10] | | [-.05, .08] | | [-.16, -.04] | | [-.13, -.00] | | [-.08, .04] | | [.12, .24] | [-.10, .02] | | [-.07, .05] | | |
|  |  | |  | |  | |  | |  | |  | |  | |  | |  | |  | |  | |  | |  | |  | | |  | |  |  |

*Note.* Values in square brackets indicate the 95% confidence interval for each correlation. The confidence interval is a plausible range of population correlations that could have caused the sample correlation (Cumming, 2014). ToP = Toilet paper. * indicates *p* < .05. ** indicates *p* < .01.
